# Small cell variant of ALK-positive anaplastic large cell lymphoma with primary subcutaneous presentation: A case report

**DOI:** 10.1097/MD.0000000000011222

**Published:** 2018-06-29

**Authors:** Tang-Her Jaing, Tsung-Yen Chang, Shih-Hsiang Chen, Yu-Chuan Wen, Wen-Yu Chuang, Chao-Ping Yang

**Affiliations:** a Division of Hematology and Oncology, Department of Pediatrics, Chang Gung Children's Hospital, Chang Gung University; b Department of Nursing, Chang Gung University and Memorial Hospital; c Department of Pathology, Chang Gung Memorial Hospital, Chang Gung University, Linkou, Taoyuan, Taiwan.

**Keywords:** anaplastic large cell lymphoma, small cell variant, transplantation

## Abstract

**Rationale:**

The rare morphological variant of anaplastic large cell lymphoma (ALCL) may pose a challenge in diagnosis, especially when presentation primarily involves skin lesions.

**Patient concerns:**

Here we describe a rare case of small cell variant of ALCL in an 11-year-old girl.

**Diagnosis:**

We performed clinical, morphological, and immunohistochemical analyses of developed cutaneous nodules.

**Interventions:**

Pathologists should consider this small cell variant in ALCL differential diagnosis, as early and correct diagnosis has important clinical implications.

**Outcomes:**

Allogeneic hematopoietic stem cell transplantation appears to be a promising treatment option for small cell variant of ALCL.

**Lessons:**

Histological diagnosis of small cell variant of ALCL is challenging among pediatricians because of its low incidence and atypical presentation. We provide a short review of the small cell variant of ALCL to facilitate the diagnosis of this difficult-to-recognize entity.

## 1 Introduction

Most cases of anaplastic lymphoma kinase-positive (ALK+) anaplastic large cell lymphoma (ALCL) exhibit a common anaplastic morphology with hallmark cells. However, a rare but well-recognized small cell ALCL variant may pose diagnostic challenge.^[1]^ Unlike adult ALCL, pediatric ALCL is commonly ALK+.^[2]^ Optimal therapy for advanced-stage pediatric ALCL is unknown.^[3]^ The small cell ALCL variant has an almost-identical presentation to ALK+ ALCL, and was first reported by Kinney et al in 1993.^[4]^ Patients with ALK+ ALCL and skin involvement represent a high-risk group that may need aggressive therapy.^[5]^ We report a case of small cell variant of ALCL, successfully treated with allogeneic hematopoietic stem cell transplantation (HSCT), and review the literature on similar cases treated by HSCT.

## 2 Case report

An 11-year-old Taiwanese girl was admitted to our hospital with fever, dyspnea, and impending respiratory failure in May 2013. Physical examination revealed an enlarged nodular lesion over the right shoulder and several smaller nodular lesions on the abdomen. A complete blood count showed leukocytosis at 19.4 × 10^9^ cells/L with 78% segmented neutrophils, 2% band form, 11% lymphocytes, and 9% monocytes. C-reactive protein level was 132.26 mg/L (normal: <5 mg/L), and serum lactate dehydrogenase level was 392 U/L (normal: 135–260 U/L). High-resolution computed tomography showed multiple lung opacities and mediastinal, cervical, and bilateral axillary lymphadenopathies. Bone marrow aspiration and biopsy revealed no lymphoma cells.

Lymph node biopsies confirmed ALK+ ALCL diagnosis. Lymphoma cells were positive for CD2, CD3, CD4, CD30, ALK1, Bcl-6, MUM1, and TIA-1, but were negative for CD20, CD5, cyclin D1, CD10, TdT, CD8, and PD1. Conventional cytogenetic analysis showed a normal karyotype. Lymph nodes also displayed a relatively small number of small-to-large hallmark cells with reniform nuclei. Because of the rate small cell ALCL variant morphology resembling classic ALCL, it was misdiagnosed initially as ALK+ ALCL. Our patient achieved complete remission 4 months after diagnosis. Treatment involved an initial course of intravenous dexamethasone and cyclophosphamide, and intrathecal administration of methotrexate, cytarabine, and hydrocortisone, followed by 3 alternating cycles of A and B regimens every 3 weeks (A: dexamethasone, high-dose methotrexate, cytarabine, etoposide, and ifosfamide; B: dexamethasone, cyclophosphamide, doxorubicin, and high-dose methotrexate). New skin lesions were later noted in the lower back, and ALCL relapse was confirmed by skin biopsy 28 months after the initial treatment. Based on these findings we reviewed the histology of subcutaneous nodules biopsy performing additional immunohistochemistry for the ALK protein which revealed positivity in some of the CD3+ small lymphocytes as well as in rare dispersed previously unrecognized atypical large cells which also turned out to be CD30+. This prompted a diagnosis of subcutaneous nodule involvement by a small cell component of an ALK+ ALCL of the composite variant. Small cell variant of ALCL was confirmed by the reviewing pathologist.

Further treatment consisted of chemotherapy with 2 courses of high-dose CHOP (cyclophosphamide 2000 mg/m^2^ [day 1], hydroxydaunorubicin 90 mg/m^2^ [day 1], oncovin 2 mg/d [day 1], prednisolone 60 mg/m^2^ [days 1–5], mesnum [150% cyclophosphamide dose]), alternating with one course of standard ESHAP ([etoposide 40 mg/m^2^ [days 1–4]; cisplatin 25 mg/m^2^ [days 1–4], cytarabine 2000 mg/m^2^ per day [day 5], and prednisolone 250 mg [days 1–4]). After completion, the patient underwent allogeneic peripheral blood stem cell transplantation from her human leukocyte antigen-identical sister in December 2015. The timeframe from initial diagnosis to transplantation was 32 months. Before transplantation, the patient had residual skin lesions suggesting a partial remission, and bone marrow biopsy revealed no residual lymphoma cells. The patient received a conditioning regimen consisting of total body irradiation (13.2 Gy in 8 fractions on days −8 to −5), and cyclophosphamide (60 mg/kg on days −3 to −2). Infused cells and CD34+ cells were 10.14 × 10^8^/kg and 10.65 × 10^6^/kg, respectively.

Graft-versus-host disease prophylaxis consisted of intravenous cyclosporine (5 mg/kg per day) beginning on day −3 and short-term methotrexate at 15 mg/m^2^ on day +1 and 10 mg/m^2^ on day +3 and +6. Rapid engraftment was obtained. Neutrophil count of >0.5 × 10^9^/L and platelet count of >20 × 10^9^/L were achieved on days 12 and 15, respectively, and complete donor chimerism was observed in a bone marrow sample obtained on day 42. After HSCT, a follow-up positron emission tomography–computed tomography showed complete metabolic remission. As of April 2018, the patient had achieved 28 months of continuous complete remission with a Lansky score of 100.

### 2.1 Pathologic findings

**Figure 1:**
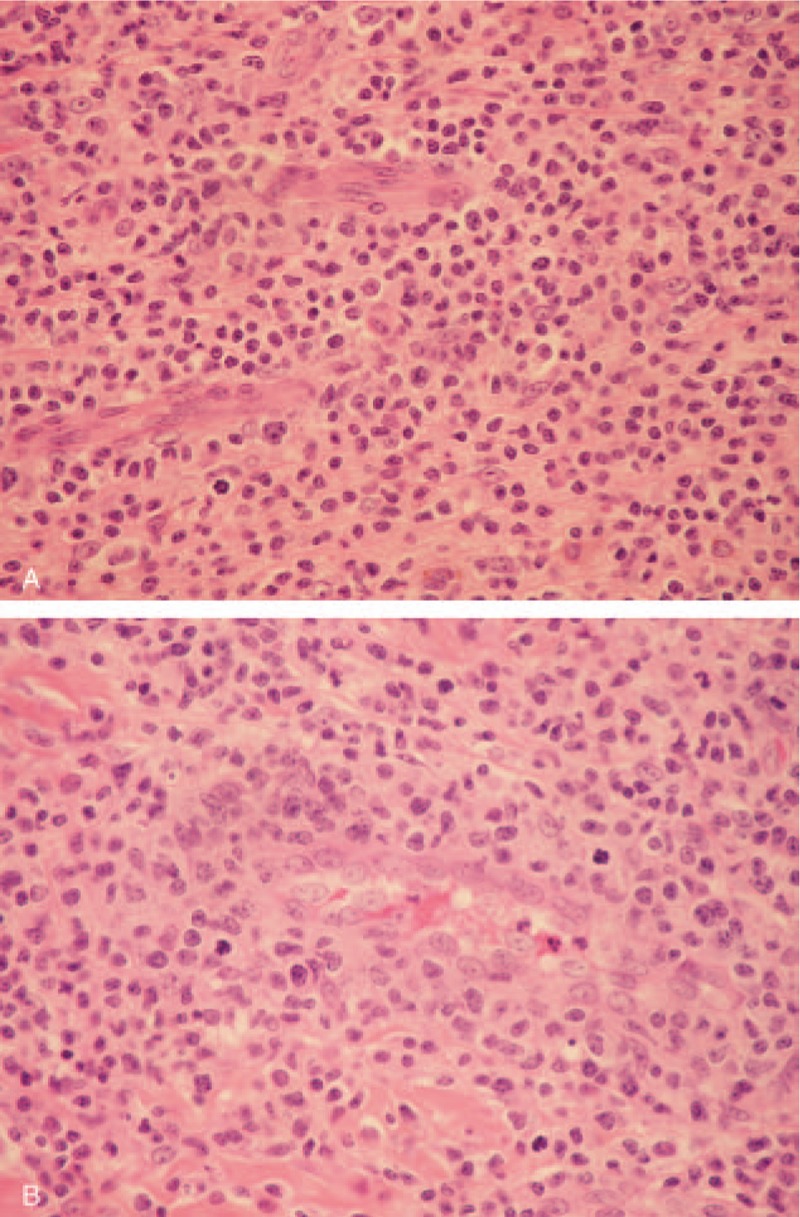
Histopathology of subcutaneous nodules. (A) H&E stain (note small-to medium-sized lymphoid cells with irregular nuclei in the majority of cells). (B) H&E stain (note the perivascular infiltrates of large lymphoid cells with anaplastic nuclei and abundant cytoplasm), ×400.

**Figure 2:**
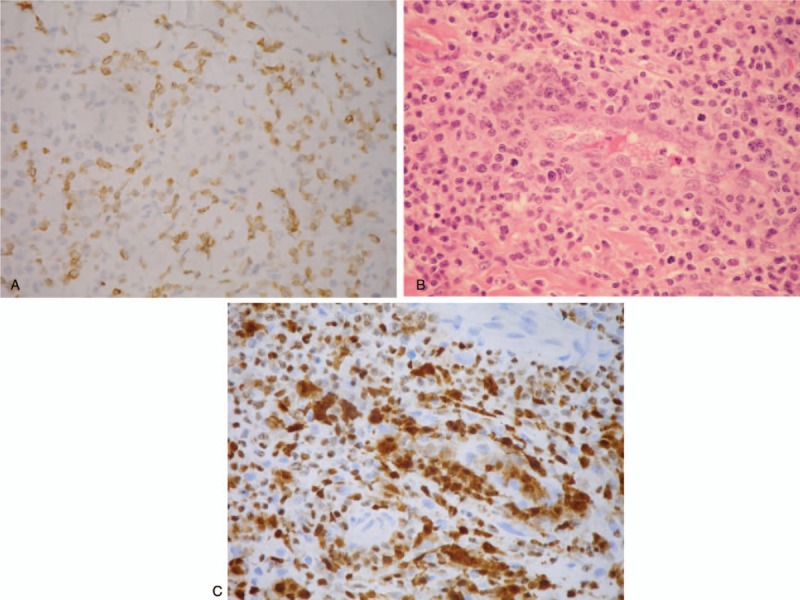
(A) CD3 stain (note the smaller tumor cells are diffusely positive for CD3, whereas the large tumor cells are only focally and weakly positive). (B) CD30 stain (note the large tumor cells showing strong CD30 positivity and the smaller tumor cells showing weak positivity). (C) ALK stain (note the large tumor cells showing strong nuclear staining for ALK and the smaller tumor cells showing weak staining), ×400.

A chest wall skin-punch biopsy demonstrated diffuse subdermal tumor cells infiltrates. Infiltrated lymphocytes consisted of small-to medium-sized cells with irregular nuclear contours, condensed chromatin, and moderately clear cytoplasm (Fig. 1A). Lymphocytes infiltrated the vascular wall with prominent rimming of individual endothelial cells (Fig. 1B). Immunohistochemical analysis showed the infiltrating cells were positive for CD2, CD3 (Fig. 2A), CD4, CD30 (Fig. 2B), ALK (Fig. 2C), Bcl-6, MUM1, and TIA-1.

## 3 Discussion

ALCL was first described in 1985 based according to its unique characteristic of cohesive proliferation of large pleomorphic cells expressing CD 30 (Ki-1).^[6]^ ALK+ ALCL has a more variable presentation but distinct phenotype. ALK+ ALCL predominantly affects young male patients and can be distinguished by histologically discernible neoplastic cells.^[1,7]^ Clinically, it frequently involves extranodal sites, including the skin, soft tissues, and viscera; although, cutaneous presentation is usually associated with identifiable lymphadenopathy and/or other tissue involvement. The small cell variant of ALCL is characterized by a predominant cytomorphology, which is unexpected for ALCL, being in the context of a small-to medium-sized hyperchromatic atypical lymphocyte. In spite of the fact that well known in its systemic form including patients with secondary cutaneous involvement, distinguishing primary cutaneous ALCL from its systemic counterpart requires notification upon pathological diagnosis.^[8,9]^

Routinely, treatment of common ALK+ ALCL includes anthracycline-based regimens, such as CHOP, with approximately 90% response rate.^[10]^ ALK protein positivity indicates excellent prognosis after standard chemotherapy.^[11]^ Although primary cutaneous ALCL tends to relapse in approximately 40% cases, long-term prognosis remains excellent because the relapses are generally cutaneous. A small cell ALCL variant presented in a nearly identical manner to the more common ALK+ ALCL, except that it is more frequently associated with leukemic involvement and pursues an aggressive clinical course.^[12–14]^ There is no clear consensus for the treatment of relapse. Several comparison studies describing approaches from a variety of reinduction chemotherapy combined in relevant publications with autologous or allogeneic HSCT have shown the majority of patients with relapsed ALK+ ALCL can be rescued.^[15]^ Allogeneic HSCT is an effective rescue therapy for high-risk ALCL relapse treatment and has acceptable toxicity.^[16]^ This clinical observation of the efficacy of allogeneic HSCT for patients with relapsed ALCL also suggests a possible graft-versus-ALCL effect.

## 4 Conclusion

The small cell variant of ALCL is rare, accounting for approximately 8% cases and lacks the anaplastic morphology commonly seen with ALCL.^[17]^ This report highlights an intriguing case of small cell variant of ALCL with initially deceiving clinical and histopathologic presentations, emphasizing the value of immunohistochemical analysis, to prevent diagnostic errors.

## Author contributions

**Conceptualization:** Tsung-Yen Chang.

**Data curation:** Tang-Her Jaing, Tsung-Yen Chang.

**Formal analysis:** Tang-Her Jaing, Shih-Hsiang Chen.

**Investigation:** Tang-Her Jaing, Wen-Yu Chuang.

**Supervision:** Chao-Ping Yang.

**Validation:** Yu-Chuan Wen, Wen-Yu Chuang.

**Visualization:** Yu-Chuan Wen, Wen-Yu Chuang.

**Writing – original draft:** Tang-Her Jaing.

**Writing – review and editing:** Tang-Her Jaing, Chao-Ping Yang.
